# Conjugative transfer of an IncA/C plasmid-borne bla_CMY-2_ gene through genetic re-arrangements with an IncX1 plasmid

**DOI:** 10.1186/1471-2180-13-264

**Published:** 2013-11-21

**Authors:** Magdalena Wiesner, Marcos Fernández-Mora, Miguel A Cevallos, Crispín Zavala-Alvarado, Mussaret B Zaidi, Edmundo Calva, Claudia Silva

**Affiliations:** 1 Departamento de Microbiología Molecular, Instituto de Biotecnología, Universidad Nacional Autónoma de México, Apartado Postal 510-3, Cuernavaca, Morelos, México; 2 Programa de Genómica Evolutiva, Centro de Ciencias Genómicas, Universidad Nacional Autónoma de México, Apartado Postal 565-A, Cuernavaca, Morelos, México; 3 Microbiology Research Laboratory, Hospital General O'Horan and Infectious Diseases Research Unit, Hospital Regional de Alta Especialidad de la Península de Yucatán, Mérida, Yucatán, México

## Abstract

**Background:**

Our observation that in the Mexican *Salmonella* Typhimurium population none of the ST19 and ST213 strains harbored both the *Salmonella* virulence plasmid (pSTV) and the prevalent IncA/C plasmid (pA/C) led us to hypothesize that restriction to horizontal transfer of these plasmids existed. We designed a conjugation scheme using ST213 strain YU39 as donor of the *bla*_CMY-2_ gene (conferring resistance to ceftriaxone; CRO) carried by pA/C, and two *E. coli* lab strains (DH5α and HB101) and two Typhimurium ST19 strains (SO1 and LT2) carrying pSTV as recipients. The aim of this study was to determine if the genetic background of the different recipient strains affected the transfer frequencies of pA/C.

**Results:**

YU39 was able to transfer CRO resistance, via a novel conjugative mechanism, to all the recipient strains although at low frequencies (10^-7^ to 10^-10^). The presence of pSTV in the recipients had little effect on the conjugation frequency. The analysis of the transconjugants showed that three different phenomena were occurring associated to the transfer of *bla*_CMY-2_: 1) the co-integration of pA/C and pX1; 2) the transposition of the CMY region from pA/C to pX1; or 3) the rearrangement of pA/C. In addition, the co-lateral mobilization of a small (5 kb) ColE1-like plasmid was observed. The transconjugant plasmids involving pX1 re-arrangements (either via co-integration or IS*Ecp1*-mediated transposition) obtained the capacity to conjugate at very high levels, similar to those found for pX1 (10^-1^). Two versions of the region containing *bla*_CMY-2_ were found to transpose to pX1: the large version was inserted into an intergenic region located where the “genetic load” operons are frequently inserted into pX1, while the short version was inserted into the *stbDE* operon involved in plasmid addiction system. This is the first study to report the acquisition of an extended spectrum cephalosporin (ESC)-resistance gene by an IncX1 plasmid.

**Conclusions:**

We showed that the transfer of the YU39 *bla*_CMY-2_ gene harbored on a non- conjugative pA/C requires the machinery of a highly conjugative pX1 plasmid. Our experiments demonstrate the complex interactions a single strain can exploit to contend with the challenge of horizontal transfer and antibiotic selective pressure.

## Background

*Salmonella enterica* subspecies *enterica* serovar Typhimurium is one of the most prevalent serovars isolated from ill humans in Mexico, with swine being the most common food-animal reservoir [1]. The appearance of strains with multiple drug resistance, including resistance to extended spectrum cephalosporins (ESC), as ceftriaxone (CRO), was reported to increase the morbidity and mortality in Yucatán, one of the poorest states in the country [2]. In order to establish the genetic composition of the emerging strains we conducted a series of investigations to determine the genetic variability of core and accessory genome compartments of the Mexican Typhimurium population. A representative collection of more than a hundred strains, derived from an integrated surveillance program including asymptomatic and ill humans, and farm-animals [1], was analyzed by multi-locus sequence typing and other molecular techniques [3,4]. In the first study, we found that the Typhimurium population from Mexico was composed of two main genotypes: ST19 and ST213. Each genotype was associated with different accessory genetic elements. The *Salmonella* virulence plasmid (pSTV) was found only in the ST19 strains, whereas the ST213 strains harbored IncA/C plasmids (pA/C), suggesting that these two genetic elements are incompatible [3,4].

In a second study, we determined that the *bla*_CMY-2_ gene conferring resistance to ESC was carried by the IncA/C plasmids harbored by ST213 strains [5]. IncA/C plasmids are recognized as having broad host ranges, but their conjugal transfer capacities are variable [6,7]. We found that most of the pA/C of ST213 strains were not conjugative under our experimental conditions; among the twenty one strains studied, only strain YUHS05-78 (YU39) was able to transfer ESC resistance to *Escherichia coli* laboratory strain DH5α [5].

The observation that in the Mexican Typhimurium population none of the ST19 and ST213 strains harbored both pSTV and pA/C led us to hypothesize that a restriction to horizontal transfer and establishment of co-residence of these plasmids, an incompatibility, existed. To address this issue we designed a conjugation scheme using ST213 strain YU39 as donor, with two *E. coli* lab strains (DH5α and HB101) and two Typhimurium ST19 strains (SO1 and LT2) as recipients. In the current study, we assessed whether the genetic background of the different recipient strains affected the transfer frequencies of pA/C, and looked for negative interactions between the transfer of pA/C and the presence of pSTV in the recipient strains.

We found that YU39 was able to transfer CRO resistance to all the recipient strains, although at low frequencies, ranging from 10^-7^ to 10^-10^. Unexpectedly, the analysis of the transconjugants showed that three different phenomena were occurring associated to the transfer of *bla*_CMY-2_: 1) the co-integration of pA/C with a co-resident IncX1 plasmid (pX1); 2) the transposition of the CRO resistance determinant *bla*_CMY-2_ from pA/C to pX1; or 3) the transfer of pA/C displaying genetic re-arrangements. In addition, the co-lateral mobilization of a small (5 kb) ColE1-like plasmid was observed. These experiments demonstrate the possibilities that a single strain can exploit to contend with the challenge of horizontal transfer and antibiotic selective pressure.

## Methods

### Typhimurium strains

To study the genetic interactions among pSTV and pA/C, we selected two strains from our previously studied Mexican Typhimurium population [4,5]. The internal review boards and ethics committees of all collaborating hospitals in the surveillance network approved the protocol, and written informed consent was collected from the guardians of all participants to obtain fecal and/or blood samples, and use the clinical and microbiologic information for scientific studies [1]. The ST213 strain YU39 was used as a pA/C donor, since this was the only strain capable of conjugal transfer [5]. This strain harbored five plasmids: the 150 kb pA/C and four plasmids of different sizes (ca. 100, 40, 5 and 3 kb), for which no information was available. We selected strain SOHS 02-2 (hereafter referred to as SO1) which contains a 94 kb pSTV and a cryptic 80 kb plasmid [4], and the reference strain LT2 which only carries the 94 kb pSTV [8], as representative strains of the ST19 genotype harboring pSTV. The pSTV of SO1 and LT2 were marked with a kanamycin resistance cassette inserted into the *spvC* gene (coding for a phosphothreonine lyase) according to the Datsenko and Wanner protocol [9]. These strains were named SO1pSTV*::Km* and LT2pSTV*::Km*, and were used as recipients in conjugation experiments (Table 1).

**Table 1:** Bacterial strains and plasmids used in this work

**Strain**	**Plasmids (kb)**	**Feature**
***Salmonella***		
YU39 (ST213)	pA/C (150), p100 (100), pX1 (40), pColE1-like (5), p3 (3)	Donor
SO1 (ST19)	pSTV*::Km* (94), p80 (80)	Recipient
LT2 (ST19)	pSTV*::Km* (94)	Recipient
***E. coli***		
DH5α		Recipient
HB101		Recipient
HB101pSTV	pSTV*::Km*	Recipient
DH5α	pA/C	Wild-type pA/C, donor
DH5α	pA/C, pSTV*::Km*	Stability assays
DH5α	pX1	Wild-type pX1
**Transconjugants**		
**SO1**		
IA4	pA/C	Re-arranged pA/C
IA5	pA/C	Re-arranged pA/C
IA9	pA/C	Re-arranged pA/C
IIA4	pA/C + pX1	pA/C and pX1 co-integrate
**HB101**		
IC2	pX1*::*CMY	pX1 with the transposed CMY region
IIC1	pX1*::*CMY	pX1 with the transposed CMY region
IIIC9	pA/C + pX1	pA/C and pX1 co-integrate
IIIC10	pX1*::*CMY	pX1 with the transposed CMY region
IVC8	pA/C + pX1	pA/C and pX1 co-integrate
**HB101pSTV** ***::Km***		
ID1	pX1*::*CMY	pX1 with the transposed CMY region
IID2	pX1*::*CMY	pX1 with the transposed CMY region
IIID8	pA/C + pX1	pA/C and pX1 co-integrate
IVD2	pA/C + pX1	pA/C and pX1 co-integrate
IVD8	pX1*::*CMY	pX1 with the transposed CMY region
**LT2**		
IIE2	pX1*::*CMY	pX1 with the transposed CMY region
IIIE4	pX1::CMY	pX1 with the transposed CMY region
IIIE9	pA/C + pX1	pA/C and pX1 co-integrate
**DH5α**		
221-1	pA/C + pX1	pA/C and pX1 co-integrate
221-10	pA/C + pX1	pA/C and pX1 co-integrate
225-1	pA/C + pX1	pA/C and pX1 co-integrate
225-7	pA/C + pX1	pA/C and pX1 co-integrate
**pX1 mutants**		
DH5α	pX1*ydgA::Tn5*	Tn*5* transposon insertion
DH5α	pX1*taxB::Km*	*taxB* site-directed mutant
DH5α	pA/C, pX1*ydgA::Tn5*	Donor
DH5α	pA/C,pX1*taxB::Km*	Donor

### Transformation of pA/C and pSTV into *E. coli* DH5α

Compatibility tests between pA/C and pSTV plasmids were performed in *Escherichia coli* laboratory strain DH5α. To obtain a DH5α harboring the two plasmids, the SO1pSTV*::Km* was transformed into DH5α and selected using kanamycin (Km; 60 μg/ml); this strain was then used a recipient for transformation with the YU39 pA/C and selected with ceftriaxone (CRO; 2 μg/ml). Transformants were evaluated for resistance to CRO and Km. Based on a previously developed PCR screening *spvC* and *traT* genes were used to track pSTV, while *repA/C* and R-7 were tested for the presence of pA/C [4,5]. Plasmid integrity was confirmed by plasmid profiling using a modified alkaline lysis procedure [10], and visualized by electrophoresis in 0.7% agarose gels subjected to 60 V for 8 hours.

### Plasmid stability tests

For the *E. coli* DH5α strain harboring both pA/C and pSTV*::Km* plasmids, stability experiments were performed (Additional file 1: Figure S1). This strain was sub-cultured for approximately 80 generations (three days) and colonies were analyzed to determine the fraction of cells in the population harboring pA/C and pSTV*::Km* plasmids. Colonies from the LB plates were picked onto LB plates containing either CRO or Km. Two randomly chosen colonies were selected in all time points for pA/C and pSTV*::Km* PCR screening with *repA/C*, R-7, *spvC* and *traT*.

### Conjugation experiments

A set of conjugation experiments was designed using YU39 as donor and five recipient strains: two Typhimurium ST19 strains SO1pSTV*::Km* and LT2pSTV*::Km*, the two laboratory *E. coli* strains DH5α and HB101, along with a transformed HB101 strain carrying the SO1pSTV*::Km* (Additional file 2: Figure S2). In addition, the YU39 pA/C was transformed into *E.coli* DH5α and the resultant strain (DH5α-pA/C) was used as a donor in the same conjugation scheme. Briefly, conjugations were performed on LB plates using a 1:10 donor to recipient mix and incubated at 37°C overnight. All the recipient strains were spontaneous resistant-mutants to rifampicin (100 μg/ml) and nalidixic acid (60 μg/ml). The overnight conjugation mix was resuspended in 2 ml of water, and dilutions were spread on LB plates containing CRO, Km and Nal as selection antibiotics. Transfer frequencies were calculated as the number of transconjugants per donor.

Some of the resultant transconjugant colonies were selected for further analysis and named using the following code: for each recipient strain a capital letter was assigned (SO1 = A, HB101 = C, HB101pSTV*::Km* = D and LT2 = E); the experiment number was coded by roman numerals from I to IV; and a colony number was assigned (Table 1). For example, transconjugant IIIC10 was the colony number 10 of the third conjugation experiment to recipient HB101.

In order to assess the integrity of the transconjugant plasmids, they were transformed into DH5α, selected with CRO, and analyzed by plasmid profiling, restriction analysis and PCR screening (see below). These transformant strains were then used as donors in a second round of conjugation, using DH5α or the original recipient strains (termed “original” second round) as recipients resistant to rifampicin and nalidixic acid. The first rounds of conjugations were performed four times, while second rounds of conjugations were performed twice.

### Cloning strategy to discover pX1 and pColE1-like

To determine the genetic identity of the non-pA/C plasmid that acquired the *bla*_CMY-2_ gene, the transconjugant plasmid of strain IC2 was restricted with 10 U of *Sau*3A, and cloned into pUC18 digested with *Bam*HI using standard methods [10]. The cloned region containing the *bla*_CMY-2_ gene was sequenced using the pUC18 *lacZ* primers (Additional file 3: Table S1), and BLAST searches were performed to detect homology with sequences in public databases (http://www.ncbi.nlm.nih.gov). The CMY region surroundings showed homology to IncX1 plasmids (pX1), and pOU1114 was selected as the reference pX1 plasmid (GenBank:DQ115387). To generate a pX1 genetic marker we designed primers to amplify the pX1 replication region (*oriX1*; Additional file 3: Table S1).

To establish the genetic identity of the 5 kb plasmid, the band was purified from the YU39 plasmid profile using Zymoclean™ Gel DNA recovery kit (ZYMO Research Corp, Irvine, CA). Libraries were constructed by digestion with *Sau*3A, and cloned into pUC18 digested with *Bam*HI using standard methods [10]. The cloned fragments were sequenced using the pUC18 *lacZ* primers (Additional file 3: Table S1), and BLAST searches were performed to detect homology with sequences in public databases (http://www.ncbi.nlm.nih.gov). The analysis of clones showed homology to *mob* regions of ColE1 plasmid family, and plasmid SN11/00Kan (GenBank:GQ470395) from Newport strain SN11 [11] was used as reference to design a PCR marker for this plasmid (*mobA*; Additional file 3: Table S1).

### Transconjugant plasmid profiles, initial PCR screening and restrictions

Plasmid profiles for transconjugant colonies were obtained by a modified alkaline lysis procedure and the Eckhardt well-lysis procedure [5]. Transconjugants were screened by PCR using primers to detect different regions of pA/C (*repA/C* and R-7), pX1 (*oriX1*) and pSTV (*spvC* and *traT*) (Additional file 3: Table S1).

For recipient strains harboring resident plasmids (SO1, LT2 and HB101pSTV*::Km*) the transconjugant plasmids carrying *bla*_CMY-2_ were transformed into DH5α using CRO as selection. These DH5α transformants were used in the second round conjugation experiments and restriction analysis. The *E. coli* DH5α transformants carrying wild-type or transconjugant pA/C were digested with 15 U of *Pst*I (Invitrogen) at 37°C for 6 hours, whereas DH5α transformants carrying wild-type or transconjugant pX1 were simultaneously digested with 10 U of *Bam*HI and *Nco*I (Fermentas) at 37°C for 3 hours. All restriction profiles were separated by electrophoresis in 0.7% agarose gels for 3 hours at 100 V. The plasmid and restriction profiles were transferred to positively charged membranes (Amersham Hybond™ N^+^) and were hybridized at 65°C with different PCR product probes labeled with α-^32^P-dCTP by standard methods [10].

### Design of pX1 PCR screening and taxC phylogeny

We used the IncX1 plasmid pOU1114 sequence as a reference to develop a PCR typing scheme for pX1 (Additional file 3: Table S1; Additional file 4: Figure S3). Six regions were selected based on their functionality: two genes involved in plasmid replication, *oriX1*, spanning the replication region, and *ydgA* coding for a type III topoisomerase, and three genes essential for the conjugation of IncX1 plasmids, *taxB* coding for the coupling protein, *taxC* coding for the relaxase, and *ddp3* coding for an auxiliary transfer protein [12-15]. The sixth region comprised an intergenic region between two conserved ORFs coding for hypothetical proteins with unknown function, designated as the 046-047 region, according to the annotation of these proteins in pOU1114. The same primer sets were used for sequencing. The *oriX1*, *taxC*, *ydgA*, *taxB*, *ddp3* and 046-047 sequences for YU39 pX1 were deposited in the GenBank under accession numbers KC954752 to KC954757, respectively. Since the *taxC* gene was recently proposed as a marker for IncX plasmids, we compared the *taxC* sequence of YU39 pX1 with those retrieved by BLAST searches (http://www.ncbi.nlm.nih.gov). Phylogenetic and molecular evolutionary analyses were conducted using *MEGA* version 5 [16].

### Generation of pX1 mutant plasmids

Several unsuccessful efforts were carried out to obtain the wild-type YU39 pX1 by selection with different antibiotics. Taking advantage of the high conjugation frequency reported for IncX1 plasmids, we obtained the YU39 pX1 by conjugation with DH5α using no antibiotic selection and PCR screening of colonies for the presence of *oriX1*. This wild-type YU39 pX1 transconjugant (DH5α-pX1) was used for hybridization experiments and to generate two mutants. To obtain a YU39 pX1 with an antibiotic selection marker, random mutagenesis with the EZ-Tn5™ < KAN-2 > Tnp (EPICENTRE®, Madison, Wisconsin) was performed following the manufacturer's recommendation. The resultant DH5α strain acquired the Tn5 transposon 398 pb upstream of stop codon in *ydgA* gene, which coded for topoisomerase III described in plasmid RP4 as a *traE* gene [17]; the plasmid was named pX1*ydgA::Tn5*. A conjugation-defective mutant was generated by the insertion of a Km resistance cassette [9] into the *taxB* gene, coding for the coupling protein, which is essential for the successful conjugation of IncX plasmids [14]. This plasmid was denominated pX1*taxB::Km*. Finally, the two YU39 pX1 mutant plasmids were transformed into DH5α-pA/C to produce DH5α strains harboring pA/C-pX1*ydgA::Tn5* and pA/C-pX1*taxB::Km* (Table 1). These strains were used as donors to test the conjugation ability of pA/C and pX1 using the conditions described in the conjugation experiments section.

## Results

### pSTV and pA/C stably co-exist in *E. coli* DH5α

Our first approach to elucidate possible negative interactions between pSTV and pA/C was to introduce both plasmids into *E. coli* laboratory strain DH5α. After transformation, the DH5α pSTV*::Km*-pA/C strain carrying both plasmids was sub-cultured for approximately 80 generations (three days) and colonies were analyzed for resistance to CRO and Km. The resistance to CRO and Km was maintained for all the colonies analyzed, and they were positive for the PCR markers of pSTV (*spvC* and *traT*) and pA/C (*repA/C* and R7). The plasmid profiles of the colonies showed the presence of both plasmids (Additional file 1: Figure S1). These results demonstrate the compatibility and stability of pSTV and pA/C in DH5α during 80 generations.

### YU39 transferred *bla*_CMY-2_ at a low frequency and the presence of pSTV had little effect

The YU39 strain carries five plasmids: the 150 kb pA/C that was previously analyzed [5], and four plasmids of different sizes (ca. 100, 40, 5 and 3 kb), for which no information was available. We determined the transfer frequency of pA/C from a ST213 strain (YU39) to two ST19 strains (SO1 and LT2) and three *E. coli* laboratory strains (DH5α, HB101 and a HB101 strain carrying the pSTV*::Km* from SO1). A schematic representation of the conjugation scheme is presented in Additional file 2: Figure S2.

YU39 transferred CRO resistance to all five recipient strains, although at low frequencies, in the range of 10^-7^ to 10^-10^ (Table 2) [5]. The lower frequencies were recorded for the two Typhimurium strains (SO1 and LT2) and HB101pSTV*::Km*, suggesting that the presence of pSTV had a slightly negative effect on the efficiency of CRO resistance transfer. For all the recipients harboring pSTV the presence of this plasmid in the transconjugants was verified by PCR (*spvC* and *traT*) and the Km resistance phenotype; a loss of pSTV was never detected. The integrity of the pSTV was observed by plasmid profiling and restriction analysis (data not shown), suggesting that this plasmid was not affected by the entrance of a new plasmid.

**Table 2:** First round conjugations for YU39 donor strain

**Recipient strain**	**Transfer frequency**^**a**^	**No. transconjugants**^**b**^	**No. pA/C positive**^**c**^	**No. pX1 positive**^**d**^	**No. ColE1**^**e **^**(% of total)**
Typhimurium SO1 (pSTV*::Km*)	10^-8^ to 10^-10^	34	34	1	27 (79)
Typhimurium LT2 (pSTV*::Km*)	10^-8^ to 10^-10^	21	2	19	1 (0.4)
*E. coli* DH5α	10^-7^ to 10^-9^	10	10	10	5 (50)
*E. coli* HB101	10^-7^ to 10^-8^	28	9	21	4 (14)
*E. coli* HB101 (pSTV*::Km*)	10^-8^	28	8	24	4 (14)

^a^The frequency was calculated as number of transconjugants per donor; the range in the orders of magnitude obtained is shown.

^b^Number of transconjugants analyzed.

^c^Number of transconjugants positive for the *repA/C* PCR marker.

^d^Number of transconjugants positive for the *oriX1* PCR marker.

^e^Number of transconjugants carrying pColE1-like.

Transconjugant colonies were examined (Table 2): all were positive for the amplification of *bla*_CMY-2_ gene (data not shown), but surprisingly, many were not positive for the amplification of the pA/C markers (*repA/C* and R-7). The plasmid profiles of the transconjugants showed the following results: 1) in SO1 and DH5α a large plasmid was found within the size range of pA/C (approximately 150-160 kb), and in most cases a small plasmid of about 5 kb was observed; and 2) in LT2, HB101 and HB101pSTV most of the colonies showed a plasmid of about 50 kb, but in a few cases a large plasmid in the range of pA/C (150-160 kb) and the small plasmid of about 5 kb were observed (Table 2). The presence of such large plasmid correlated with those transconjugants positive for the pA/C markers, while the transconjugants harboring the 50 kb plasmid were negative. These results suggested that the 50 kb plasmid was a non-pA/C plasmid that acquired the *bla*_CMY-2_ gene.

### The YU39 CMY region from pA/C transposed to a co-resident pX1 and was transferred to LT2 and HB101 recipients

To determine the genetic identity of the 50 kb transconjugant plasmids, the plasmid of a HB101 transconjugant (IC2) was restricted, cloned and selected with CRO. The region surrounding the CMY region showed homology to sequences of IncX1 plasmids (pX1). We selected pOU1114 as reference pX1 plasmid (GenBank: DQ115387). The sequence of the cloned region containing the *bla*_CMY-2_ gene showed that it was inserted into an intergenic region between two ORFs (046-047) annotated as hypothetical proteins. We designed primers to amplify the pX1 replication region (*oriX1*), and all the 50 kb transconjugant plasmids were positive, confirming that these were pX1. Hybridizations using the *oriX1* probe on the plasmid profile of the YU39 donor strain showed that the 40 kb band corresponded to the pX1. These results showed that in YU39 the CMY region moved from pA/C to pX1, and then was transferred to LT2 and HB101.

Eight pX1 transconjugants carrying the CMY region (pX1*::*CMY) were selected for detailed analysis (Table 3). We developed a PCR typing scheme for six regions covering pX1 (Additional file 4: Figure S3). The pX1 PCR screening of the transconjugants showed that four markers were present in all the transconjugants (*oriX1*, *taxC*, *taxB* and *ddp3*). Three transconjugants were negative for the 046-047 section, and one was negative for *ydgA* gene (Table 3).

**Table 3:** **Description of the pX1 *:: *** **CMY transconjugants obtained from the YU39 donor**

**Recipient**	**pX1 *:: *** **CMY colony**	**pX1 PCR typing**						**CMY region**^**a**^	**Insertion region**^**b**^	**Second round conjugation**^**c**^	
		***oriX1***	***ydgA***	***taxB***	***taxC***	***ddp3***	**046-047**			**Original**	**DH5α**
HB101	IC2	+	+	+	+	+	-	Large	046-047	10^-1^	1 to 10^-2^
	IIC1	+	-	+	+	+	+	Short	ND	10^-1^	10^-1^ to 10^-2^
	IIIC10	+	+	+	+	+	+	Short	*stbE*	10^-1^	10^-1^ to 10^-2^
HB101 (pSTV*::Km*)	ID1	+	+	+	+	+	-	Large	046-047	10^-1^	1 to 10^-1^
	IIID2	+	+	+	+	+	-	Large	046-047	10^-2^ to 10^-4^	10^-4^ to 10^-7^
	IVD8	+	+	+	+	+	+	Short	*stbE*	10^-1^ to 10^-2^	10^-1^
LT2	IIE2	+	+	+	+	+	+	Short	*stbE*	10^-1^ to 10^-5^	10^-1^
(pSTV*::Km*)	IIIE4	+	+	+	+	+	+	Large	ND	10^-4^ to 10^-7^	1 to 10^-1^

^a^The long CMY region includes from IS*Ecp1* to hypothetical protein 0093, and the short region includes from IS*Ecp1* to *sugE* (see text for details).

^b^Section of pX1 where the CMY region was inserted (see text for details). ND, not determined.

^c^Transconjugants were challenged for a second round of conjugation using as recipients the original recipient strain (original) or DH5α. The frequency was calculated as number of transconjugants per donor; the range in the orders of magnitude obtained is shown.

**Figure 1:**
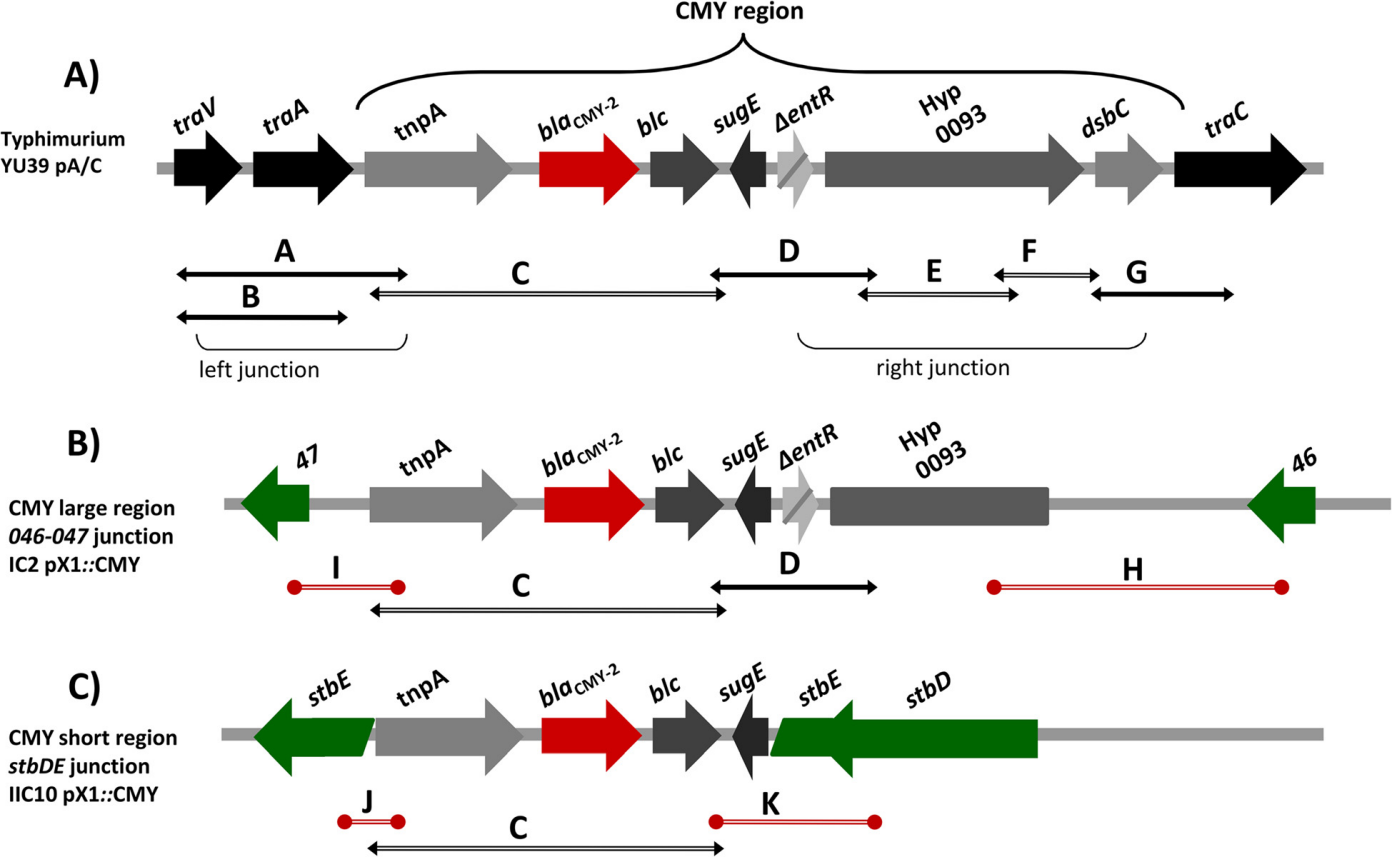
**Schematic diagram of the CMY regions of Typhimurium strain YU39 and pX1*::*CMY transconjugants.** Panel **A)** shows a schematic diagram of the CMY region in the pA/C plasmid of strain YU39 [5]. Panel **B)** depicts a large CMY region inserted into the intergenic region between 046 and 047 genes for IC2 transconjugant. Panel **C)** shows a short CMY region inserted into *stbE* gene for IIIC10 transconjugant. The PCR amplifications designed to assess the extension of the CMY regions are indicated by double arrowheads under the diagrams. The PCRs used to determine the pX1 CMY junctions are indicated by bars with circles.

To address the extent of the CMY region transferred from pA/C to pX1 we used the PCR typing scheme developed in our previous studies (Figure 1A). Four of the pX1 transconjugants were positive for six of the seven genes present in the complete CMY region of pA/C (c. a. 12 kb), spanning from IS*Ecp1* to hypothetical protein 0093 (according to pSN254 annotation; GenBank:NC_009140); while the other four displayed a short version of the CMY region (c. a. 3 kb) including only IS*Ecp1*, *bla*_CMY-2_, *blc* and *sugE* (Figure 1B and Figure 1C).

**Figure 2:**
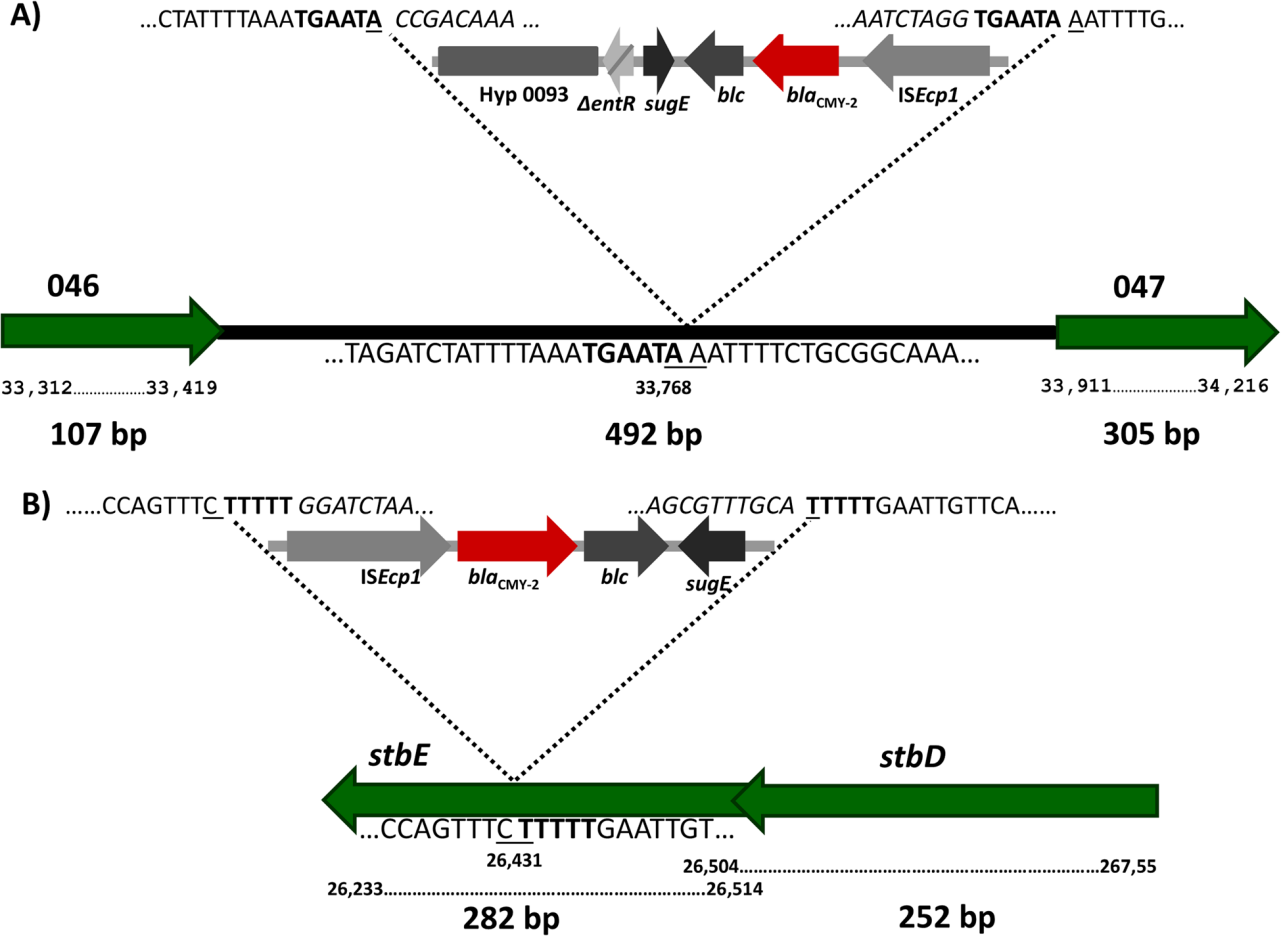
**Schematic representation for the insertion sites for the CMY region into the pX1 backbone.** Panel **A)** depicts the insertion of the large CMY region into the intergenic region between 046 and 047 hypothetical proteins. Panel **B)** shows the insertion of the short CMY region into *stbE*. The numbers under the solid black arrows correspond to nucleotide numbers in the annotation of the reference pX1 pOU1114 (GenBank: DQ115387). The surrounding nucleotide sequences at the insertion points are shown. Underlined letters mark the insertion site of the CMY region, and below the nucleotide number in the annotation of pOU1114. The CMY region sequence is indicated in italics, and the duplicated sequences generated during the transposition events are highlighted in boldface.

For the characterization of pX1 transconjugants IC2, ID1 and IIID2, that were negative for the 046-047 region, we used a combination of primers from the CMY region along with the primers for 046-047 to determine if this was the site of insertion (Figure 1B; PCRs H and I). We successfully established that the IC2, ID1 and IIID2 transconjugants were positive for the CMY-046-047 junction (Table 3). Sequencing of these PCR products showed the exact insertion site for these pX1 transconjugants harboring a large CMY region. The schematic representation of the insertion of the CMY region into 046-047 in IC2 is presented in Figure 2A. Mapping according to the pOU1114 annotation revealed that the insertion site was in nucleotide 33,768. A repeat sequence of six nucleotides (TGAATA) flanking the CMY region was detected, corresponding to nucleotides 33,763 to 33,768 of pOU1114. We discovered that the hypothetical protein 0093 was truncated at nucleotide 4,168 removing 1,318 nucleotides of the complete ORF.

On the other hand, transconjugant IIIC10, positive for the six pX1 PCR markers and harboring a short version of the CMY region, was selected to determine the site of CMY insertion, using the same approach as for IC2. The cloning and sequencing of the CMY region showed that in this plasmid the CMY region was inserted into the *stbE* gene, which is part of the *stbDE* operon coding for the toxin-antitoxin segregation system of pX1 [13]. Based on this result, we designed primers to amplify the *stbDE* operon, and these were used along with the short CMY region primers to test the other pX1*::*CMY transconjugants (Figure 1C; PCRs J and K). Positive results for pX1*::*CMY transconjugants IIIC10, IVD8 and IIE2 demonstrated the presence of the CMY-*stbDE* junction (Table 3). Careful revision of the sequences showed that the target site of insertion was nucleotide 26,431 and the signature left by the transposition event was a five bp repeat sequence (TTTTT) spanning from nucleotides 26,432 to 26,436 in the pOU1114 sequence annotation. In these short CMY regions the *sugE* ORF (441 pb) was truncated at nucleotide 367 (Figure 2B). The insertion site for pX1*::*CMY transconjugants IIC1 and IIIE4 could not be determined, despite several efforts carried out using the above mentioned approaches (Table 3).

**Figure 3:**
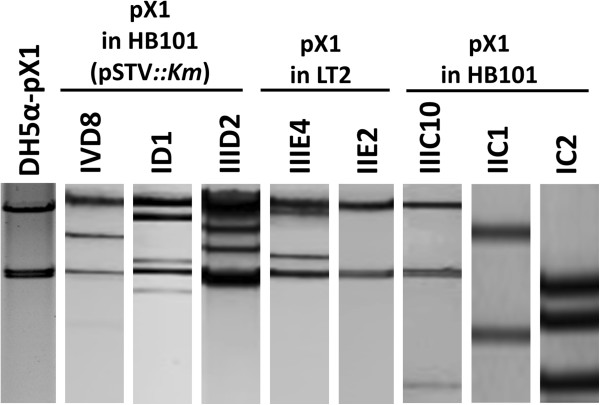
**Representative restriction profiles for pX1 + CMY transconjugants.** Double digestions with *BamH*I-*Nco*I were generated for the wild-type YU39 pX1 (DH5α-pX1) and representative transconjugant plasmids. The nomenclature of the transconjugants is shown in Table 3.

Restriction profiles for the eight pX1 transconjugant plasmids using *Bam*HI-*Nco*I enzymes displayed marked differences in comparison with the profile of wild-type YU39 pX1 transformed into DH5α (DH5α-pX1; Figure 3). These differences could be related to distinct insertion sites of the CMY region and other re-arrangements within pX1 and await further studies.

### TheYU39 pX1 mobilized *in cis* the *bla*_CMY-2_-carrying pA/C to DH5α and few of the other recipient strains

During the PCR screening of the pX1 transconjugants we discovered that all the pA/C transconjugants from DH5α were positive for the six pX1 markers. The few pA/C positive transconjugants from HB101 were also positive for the six pX1 markers, with the exception of transconjugant IIID8 which was positive only for *oriX1* and *ydgA* (Table 4). In the SO1 recipient only pA/C positive transconjugants were obtained (Table 2); although the PCR screening for pX1 in the 34 transconjugants showed that only IIIA4 was positive (Table 2 and Table 4).

**Table 4:** Description of the pA/C and pA/C + pX1 transconjugants obtained from the YU39 donor

		**pX1 PCR typing**						**Second round conjugation**^**a**^	
**Recipient**	**pA/C colony**	***oriX1***	***ydgA***	***taxB***	***taxC***	***ddp3***	**046-047**	**Original**	**DH5α**
SO1	IA4	-	-	-	-	-	-	0^b^	0
	IA5	-	-	-	-	-	-	0	0
	IA9	-	-	-	-	-	-	0	0
	IIIA4	+	+	+	+	+	+	10^-8^	1 to 10^-2^
DH5α	221-1	+	+	+	+	+	+	10^-1^ to 10^-3^	
	221-10	+	+	+	+	+	+	1 to 10^-2^	
	225-1	+	+	+	+	+	+	1 to 10^-2^	
	225-7	+	+	+	+	+	+	10^-1^ to 10^-2^	
HB101	IIIC9	+	+	+	+	+	+	10^-2^ to 10^-4^	1 to 10^-2^
	IVC8	+	+	+	+	+	+	10^-2^	10^-1^ to 10^-2^
HB101	IIID8	+	+	-	-	-	-	10^-5^	10^-5^ to 10^-7^
(pSTV*::Km*)	IVD2	+	+	+	+	+	+	1 to 10^-2^	0
LT2 (pSTV*::Km*)	IIIE9	-	-	-	-	-	-	0	10^-4^ to 10^-7^

^a^Transconjugants were challenged for a second round of conjugation using as recipients the original recipient strain (original) or DH5α. The frequency was calculated as number of transconjugants per donor; the range in the orders of magnitude obtained is shown.

^b^No transconjugants were detected under the detection level (<10^-10^).

**Figure 4:**
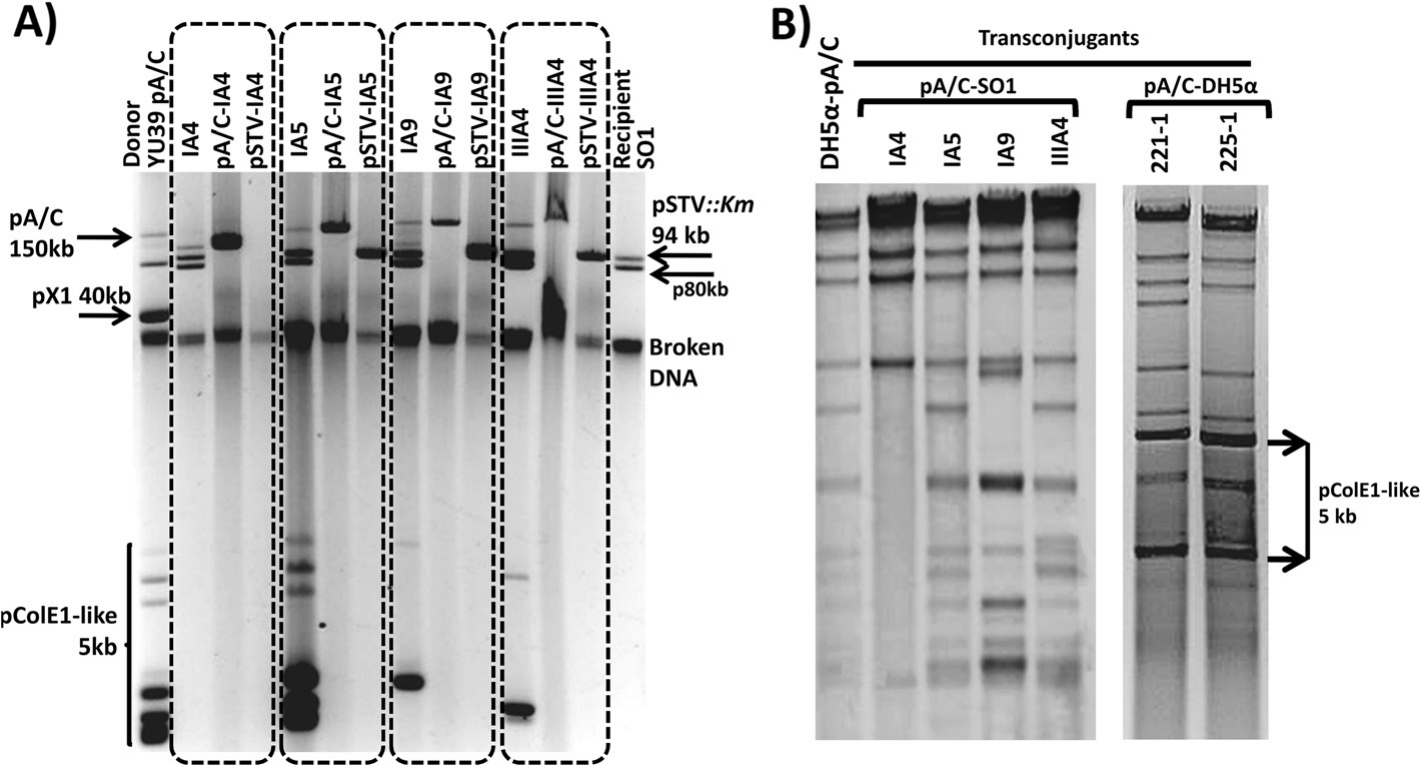
**Examples of pA/C transconjugants recovered in SO1 pSTV*::Km *and DH5α.** Panel **A)** shows the plasmid profiles of four different transconjugants in SO1 marked within dotted rectangles. The donor YU39 pA/C and the recipient SO1pSTV*::Km* strains are in the first and last lanes, respectively. Within each dotted rectangle, in the first lane are the SO1 transconjugants; in the second and third lanes the DH5α transformants for the pA/C and pSTV of each transconjugant are shown. Panel **B)** displays examples of *Pst*I restriction profiles of pA/C transconjugants of SO1 and DH5α compared with wild-type YU39 pA/C (DH5α-pA/C).

*Pst*I restriction profiles for the thirteen pA/C transconjugants selected for detailed analysis (Table 4) showed that in some cases a distinct profile was generated in comparison with that of the wild-type YU39 pA/C transformed into DH5α (DH5α-pA/C). Examples of the plasmid (Figure 4A) and *Pst*I restriction profiles are shown (Figure 4B).

**Figure 5:**
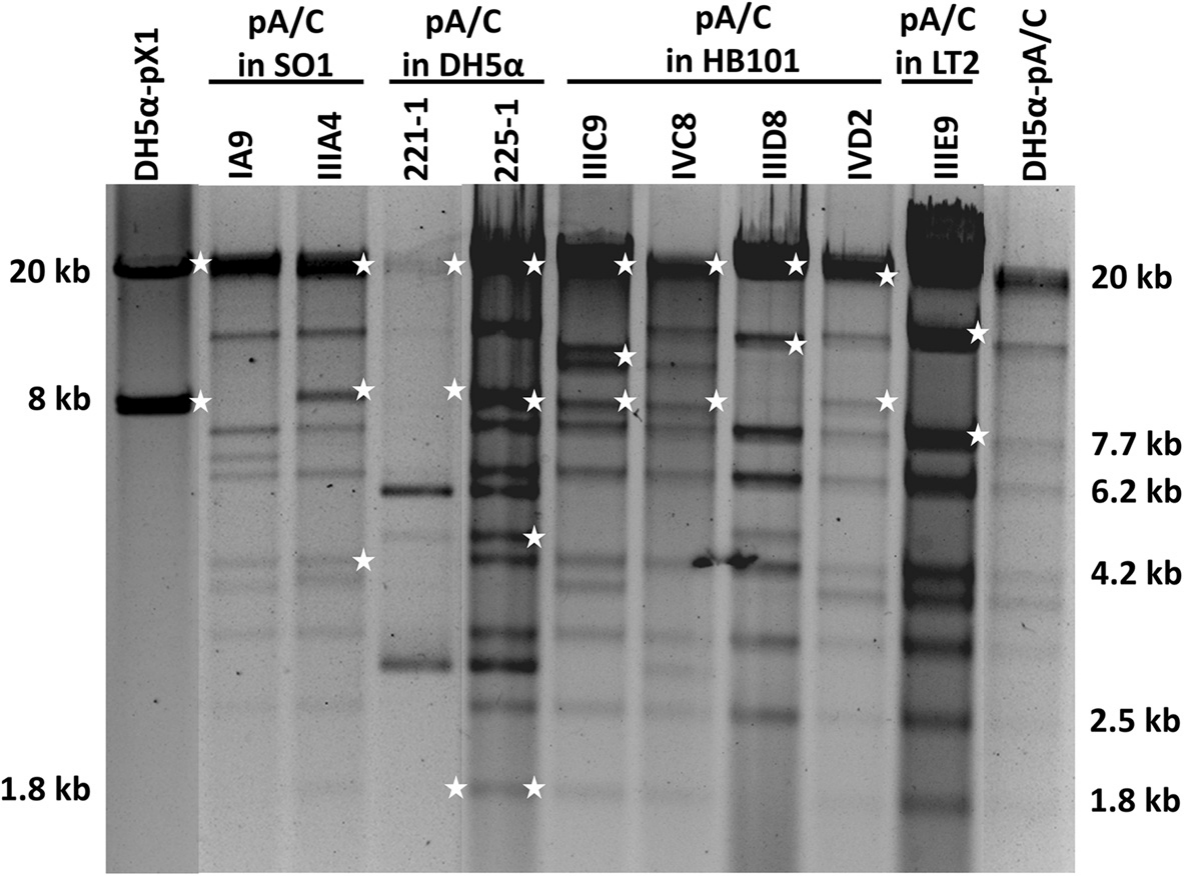
**Representative restriction profiles for pA/C transconjugants.** Double digestions with *BamH*I-*Nco*I were generated for the wild-type YU39 pX1 (DH5α-pX1) and pA/C (DH5α-pA/C) and representative pA/C transconjugant plasmids. The nomenclature of the transconjugants is shown in Table 4. White stars at the right side of the bands indicate positive hybridizations signals with the pX1 probe.

In order to detect the presence of pX1 in the pA/C transconjugants, *Bam*HI-*Nco*I restriction digests were performed, since these enzymes were used to analyze pX1. Most of the bands of the wild-type DH5α-pA/C were visible in the restriction profiles of the transconjugants, but new bands were also evident (Figure 5). When hybridized with the complete pX1 as probe, positive signals in bands corresponding with the pX1 restriction profile were obtained in most of the cases (Figure 5). SO1 transconjugant IA9 was negative for the pX1 hybridization, in agreement with the pX1 PCR screening; whereas the LT2 transconjugant IIIE9 produced hybridization signals, suggesting that this plasmid contained regions of pX1 not included in the PCR scheme (Figure 5 and Table 3). These results indicate that, with the exception of IIID8 and IIIE9, in most of the cases complete pX1 and pA/C formed co-integrates that were not resolved in the recipient strain. In any case, this finding indicates a type of *cis*-mobilization, in which the mobilized replicon is fused to a conjugative plasmid, which supplies both *oriT* and the *tra* functions [18].

We speculated that co-integration points between pA/C and pX1 could be the intergenic region 046-047 or *stbE*, as for some pX1*::*CMY transposition events. However, the amplification for these regions did not show evidence of insertions. In addition, the positive amplification of the right and left junctions of the CMY region (Figure 2a) showed that this region remained inserted into the pA/C backbone, suggesting that the regions involved in pA/C + pX1 co-integration were not those detected in pX1*::*CMY.

### The pX1::CMY and pA/C + pX1 plasmids transfer at high frequencies

The variability exhibited by the restriction profiles of the transconjugant plasmids (Figure 3, Figure 4B and Figure 5) led us to ask whether these plasmids were still able to conjugate. For this purpose, the transconjugant plasmids were electroporated into DH5α and challenged for conjugation in a “second round”. DH5α was used as recipient strain along with the original recipient in which the transconjugant plasmid was obtained, and to distinguish these second round experiments the terms “DH5α” and “original” were used, respectively.

The second round conjugation frequencies in most of the eight pX1*::*CMY were extremely high, on the order of 10^-1^ (Table 3). These frequencies were three to seven orders of magnitude higher than the frequencies recorded in the first round of conjugations (Table 2). In some cases the conjugation frequency was higher for the DH5α receptor than for the original receptor, the most drastic effect was observed for LT2 transconjugant plasmid of IIIE4 (Table 3).

The four pA/C that were negative for the pX1 PCR markers were unable to transfer CRO resistance in a second round of conjugation, whereas the eight pA/C + pX1 that were positive for all the pX1 PCR markers increased their second round conjugation frequencies by one to seven orders of magnitude (Table 4). An exception was the SO1 IIIA4 plasmid, in which the original second round conjugation retained its first round low frequency, suggesting the existence of restrictions for the entrance pA/C + pX1 to SO1. This result was later related to the observation that in SO1 most of the pA/C transconjugants were negative for pX1 markers (Table 2).

### The SO1 pA/C transconjugants were non-conjugative and display plasmid re-arrangements

The analysis of the pA/C transconjugants from SO1 (with the exception of IIIA4) showed three salient features. First, the PCR and hybridization experiments showed that they did not contain genetic material from pX1 (Table 4 and Figure 5). Second, their restriction profiles were in most cases distinct from that of the wild-type pA/C (Figure 4B and Figure 5), suggesting that different genetic re-arrangements were occurring within these plasmids. And third, when challenged in a second round experiment they were non-conjugative or below the detection level (<10^-10^; Table 4). We suppose that the re-arrangements presented by these plasmids could have arisen by recombination within each pA/C or by interaction with pX1 within the donor strain, although pX1 was not observed in SO1. The most plausible hypothesis is that co-integrates of pA/C and pX1 plasmids were formed, but were not stable in SO1 and the pX1 was lost. Incompatibility with the cryptic p80 plasmid present in the SO1 recipient could not be ruled out, to explain the lack of pX1 in these transconjugants.

### The *bla*_CMY-2_ gene carried in a non-conjugative pA/C was transferred by the highly conjugative pX1

We had previously reported that although YU39 was able to transfer CRO resistance to a DH5α recipient, a transformant DH5α strain with the YU39 pA/C (DH5α-pA/C) was unable to transfer CRO resistance to a DH5α recipient [5]. In the present study we confirmed this result and found that DH5α-pA/C was also unable to transfer CRO resistance to any of the other strains used as recipients (data not shown). Based on these results, we hypothesized that pA/C was not conjugative and that it was co-mobilized by the highly conjugative pX1. To test this hypothesis, conjugation experiments were designed using two pX1 mutants. The pX1*ydgA::Tn5* was obtained by random mutagenesis to introduce a Km resistance marker into pX1, and pX1*taxB::Km* which was created by directed mutagenesis to knockout *taxB*, coding for the coupling protein, indispensable for pX1 conjugation [14]. Each of these plasmids were introduced by transformation to DH5α-pA/C strains and challenged for conjugative transfer.

The pX1*ydgA::Tn5* displayed a very high conjugation frequency (10^-1^; Table 5), as for many of the pX1*::*CMY hybrids (Table 3). The conjugation frequency for the DH5α strain carrying pA/C and pX1*ydgA::Tn5* was 10^-1^ when only Km was used for transconjugant selection, but dropped to 10^-7^ when CRO or Km-CRO were used for selection (Table 5). The PCR analysis of the latter transconjugants showed that in all the cases the plasmids were positive for both pA/C and pX1 markers, indicating that pA/C + pX1 were recovered, in agreement with the expectations for a DH5α receptor (Table 4 and Table 5). On the other hand, the DH5α strain carrying pA/C and pX1*taxB::Km* was unable to transfer any of the plasmids under Km or CRO selection, indicating that in the presence of a conjugative-defective pX1 plasmid the pA/C was unable to transfer. In conjunction, these results support our hypothesis that pX1 contributed the conjugation machinery for pA/C transfer.

**Table 5:** Conjugation experiments for pA/C and pX1 mutants using DH5α as recipient

**DH5α donor strain**	**Selection**	**Transfer frequency**^**a**^	**No. transconjugants**^**c**^	**No. pA/C positive**^**d**^	**No. pX1 positive**^**e**^
pX1y*dgA::Tn5*	Km	10^-1^	8	0	8
pX1*taxB::Km*	Km	0^b^	0	0	0
pA/C, pX1*ydgA::Tn5*	Km	1 to 10^-2^	4	0	4
	CRO	10^-5^ to 10^-7^	6	6	6
	CRO-Km	10^-5^ to 10^-7^	12	12	12
pA/C, pX1*taxB::Km*	Km	0	0	0	0
	CRO	0	0	0	0
	CRO-Km	0	0	0	0

^a^The frequency was calculated as number of transconjugants per donor; the range in the orders of magnitude obtained is shown.

^b^No transconjugants were detected under the detection level (<10^-10^).

^c^Number of transconjugants analyzed.

^d^Number of transconjugants positive for the *repA/C* PCR marker.

^e^Number of transconjugants positive for the *oriX1* PCR marker.

We calculated that the transposition and co-integration events occurred within YU39 at frequencies between 10^-6^ and 10^-9^, based on the difference between the conjugation frequency of pA/C + pX1 and pX1*::*CMY transconjugants (10^-7^ and 10^-10^; Table 2 and Table 4) compared with that of pX1*ydgA::Tn5* (10^-1^; Table 5). It is worth noting that these conjugation experiments involving a DH5α donor carrying pA/C and pX1 produced the same results observed as when the YU39 wild-type strain was used as donor, indicating that the interaction between these plasmids did not require additional elements from the YU39 genome.

### pColE1-like was preferentially trans-mobilized along with pA/C

To determine the genetic identity of the 5 kb plasmid the band was purified, digested and cloned. The sequences from the cloned fragments showed homology to the replication and *mob* genes of ColE1 plasmids, indicating that the 5 kb was a ColE1-like plasmid (pColE1-like). PCR screening using specific primers to amplify the pColE1-like *mobA* region (Additional file 3: Table S1) showed that YU39 and all the transconjugants displaying the 5 kb band were positive. The *mobA* PCR product was employed as a probe to hybridize YU39 and transconjugants plasmid profiles. These hybridizations confirmed the identity of the 5 kb band and, in addition, showed that the pColE1-like was not involved in the formation of pA/C + X1 co-integrates or pX1*::*CMY.

The pColE1-like was mobilized *in trans* with all the DH5α pA/C + X1, with most of the SO1 pA/C transconjugants and with a few pX1*::*CMY transconjugants (Table 2), indicating stable co-existence with pA/C and pX1, and with pSTV when present.

### The YU39 pX1 is closely related to other *E. coli* and *Salmonella* pX1

**Figure 6:**
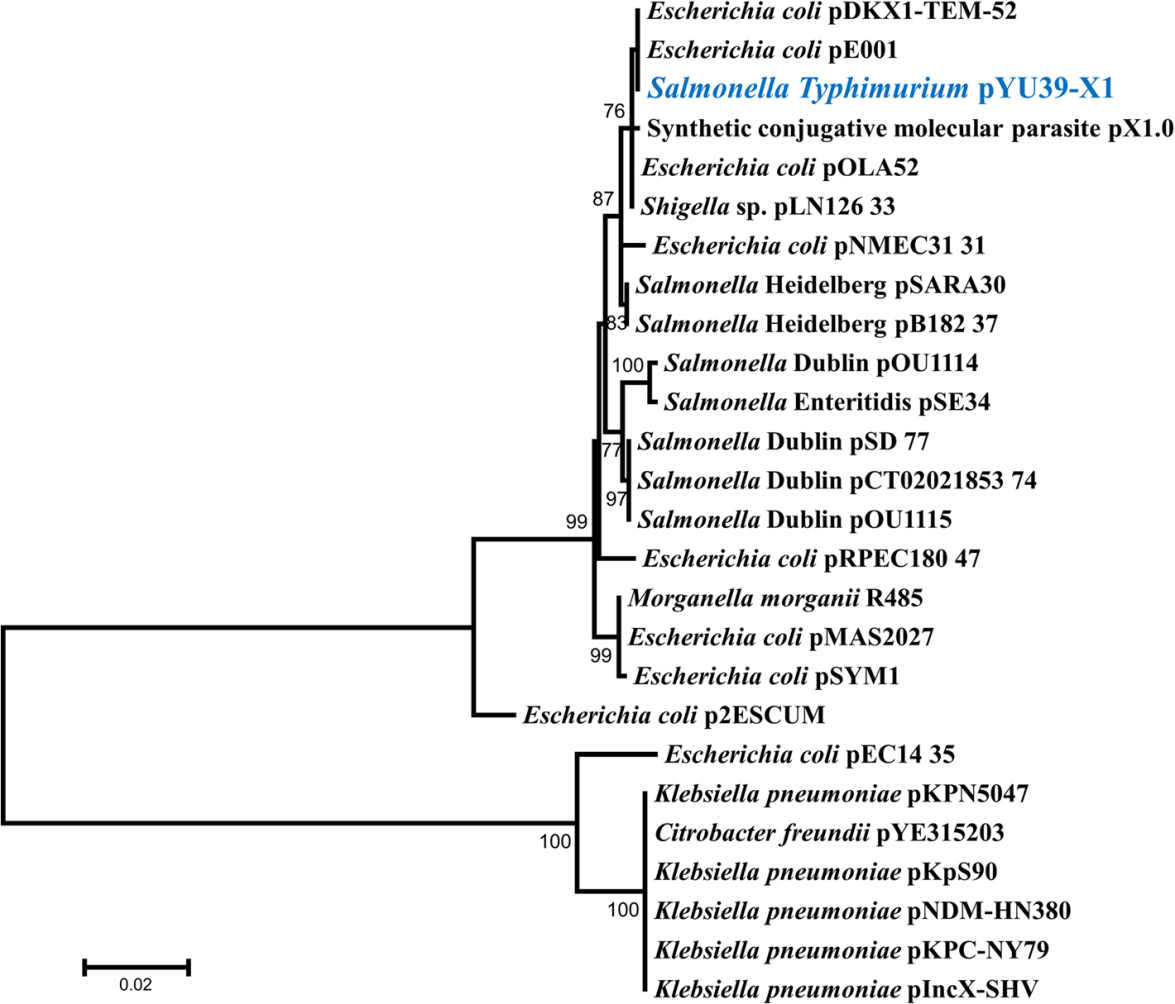
**Genetic relationships of YU39 pX1 and other IncX plasmids.** The dendrogram was constructed using the Maximum Likelihood method based on the HKY + G model with 500 bootstrap replicates. Bootstrap values greater than 75 are shown next to the nodes.

The nucleotide sequences for the six regions selected for the pX1 PCR screening showed that the YU39 pX1 was highly similar to other pX1 plasmids. In a recent study, Johnson et al. proposed the use of the *taxC* sequence as a genetic marker to compare IncX plasmids [19]. The phylogenetic inference obtained by the comparison of the *taxC* partial sequence of the YU39 pX1 with those of IncX plasmids showed that it was closely related to other *E. coli* and *Salmonella* IncX1 plasmids (Figure 6). Similar phylogenetic reconstructions were observed for the other five YU39 pX1 sequences (data not shown).

## Discussion

**Figure 7:**
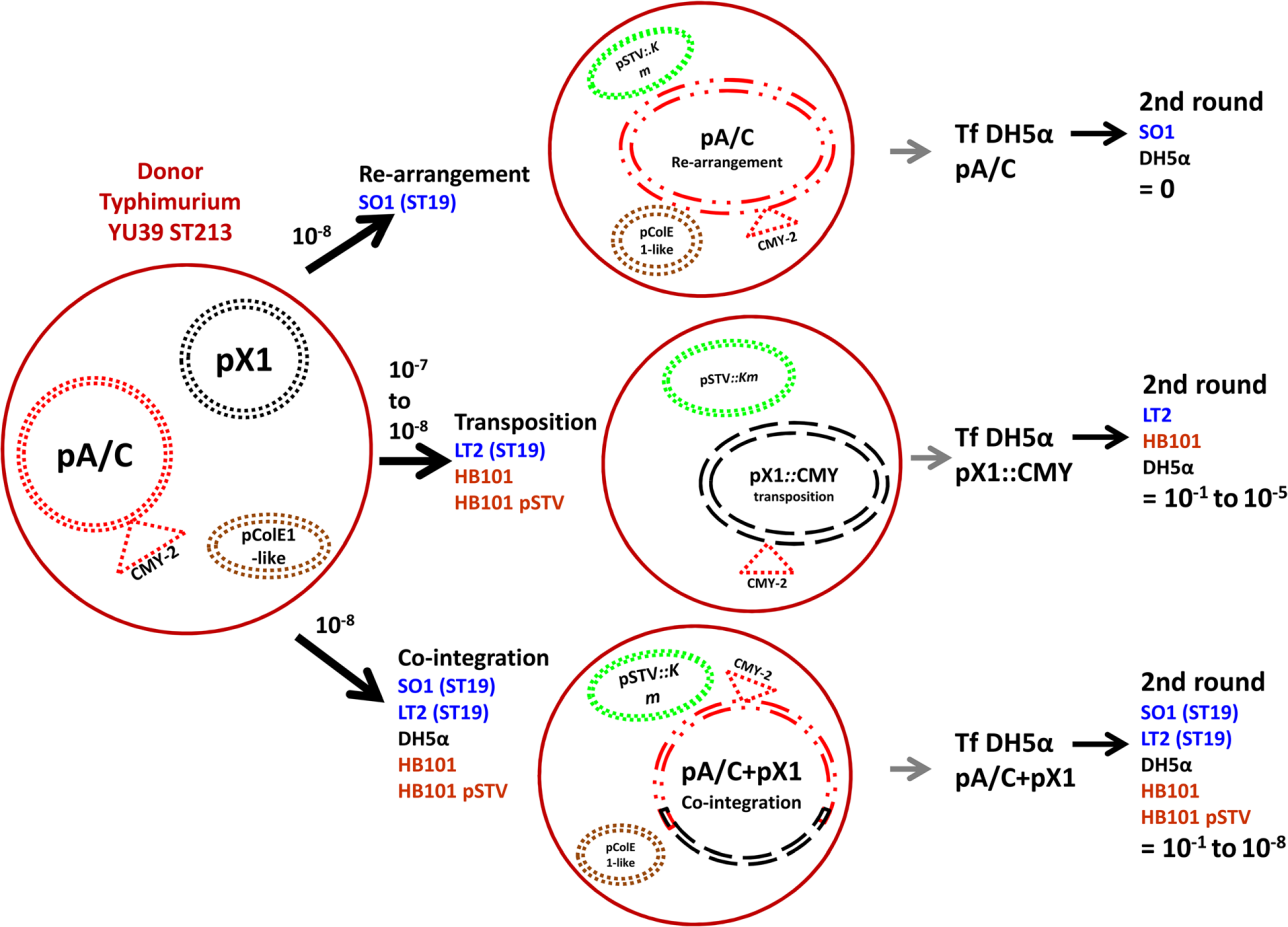
**Schematic representation of the outcome of the conjugative transfer of the YU39 IncA/C plasmid-borne *bla***_**CMY-2 **_**gene to different recipient strains.** On the left side is the donor strain YU39 harboring pA/C, pX1 and pColE1-like plasmids. In the middle, The first round conjugation frequencies are indicated above the black arrows along with the recipient strains involved in the different phenomena. The three different types of transconjugants observed for the first round are depicted. The pSTV and pColE1-like plasmids are shown, although they were not present in all the transconjugants. Electroporation step to DH5α for the transconjugants plasmids is represented with the grey arrows. Following are the range of the second round conjugation frequencies in the “original” and DH5α strains (see text for details).

In this paper we describe diverse interactions among pA/C, pX1 and pColE1-like plasmids within a single strain. When strain YU39 was challenged for conjugation different phenomena were recorded, depending on the recipient strain. When *bla*_CMY-2_ was transferred, three genetic interactions occurred at very low frequencies: 1) the co-integration of pA/C and pX1; 2) the transposition of the CMY region from pA/C to pX1; or 3) the rearrangement of pA/C. Moreover, the *trans*-mobilization of the pColE1-like plasmid occurred in most of the cases. The general outcome of these processes was the transfer of the *bla*_CMY-2_ gene from a non-conjugative pA/C to a highly conjugative pX1 plasmid. Both the resultant pA/C + pX1 and pX1*::*CMY plasmids acquired the capacity to spread ESC resistance at very high levels by conjugation (Figure 7). This mode of *cis*-mobilization and transfer of the *bla*_CMY-2_ gene has not been previously reported.

### The outcome of conjugation was dependent on the recipient stain

Distinct interactions occurred among the pA/C, pX1 and pColE1-like plasmids within the donor YU39 strain, albeit at very low frequencies. One of the most surprising results was that these interactions were differentially “sampled” in a recipient-dependent manner. We would expect to find differences between Typhimurium and *E. coli* due to the induced mutations carried by the *E. coli* laboratory strains (such as *recA*-). However, in general terms *E. coli* and Typhimurium strains shared similar results: DH5α and SO1 received and harbored pA/C and pColE1-like, while LT2 and HB101 received and harbored pX1. The study of the genetic interactions among pA/C, pX1 and pColE1-like was beyond the scope of this study. The interactions between relaxases and the pX1 coupling protein will be addressed in future studies. Moreover, the complete sequencing of the YU39 genome and representative plasmid re-arrangements is underway.

### Co-integration of the non-conjugative pA/C with the highly conjugative pX1

IncA/C plasmids encoding multi-drug resistance have been extensively studied and are known for their diversity and plasticity [6,19,20]. Several studies have determined that pA/C plasmids are prevalent in multi-drug resistant *E. coli* and *Salmonella enterica* serovars [7,21-23]. Currently, there are over twenty sequenced pA/C, and the acquisition of new antibiotic resistance determinants have been reported [20,24,25]. Although these plasmids have been found in a wide range of *Enterobacteriaceae* and a molecular signature-analysis has shown a broad evolutionary host range [26], the evidence for their conjugation ability remains controversial. Welch *et al*. analyzed the pA/C transfer ability for several *Salmonella* serovars, and reported low to moderately high conjugation frequencies (10^-3^ to 10^-7^) along with non-conjugative plasmids [7]. However, the transconjugants obtained were not analyzed to confirm self-transmissibility. Poole *et al*. studied the conjugative transferability of pA/C containing or lacking the *bla*_CMY-2_ gene in *Salmonella* Newport, concluding that plasmids encoding *bla*_CMY-2_ were rarely transferred compared with high conjugation frequencies when *bla*_CMY-2_ was absent [27]. When pA/C was the only replicon no transconjugants were detected, and much higher conjugation frequencies, between 10^-2^ and 10^-5^, were observed only when other plasmids were present and co-transferred, suggesting that other replicons are necessary for pA/C transfer [27]. Call *et al*. also reported the failure of self-conjugation for *E. coli* and Newport *bla*_CMY-2_ positive pA/C [28]. Several studies have suggested that the failure of transferability of *bla*_CMY-2_ positive pA/C was due to the insertion of this gene within one of the *tra* regions [7,27,28]. However, pAR060302 is an example of a *bla*_CMY-2_ bearing pA/C for which transfer frequencies as high as 10^-3^ are recorded [28].

In the present study, we report that the transferability of YU39 pA/C depends on the presence of YU39 pX1. Our results support the notion that the pA/C (with or without *bla*_CMY-2_) in the Mexican Typhimurium population are not self-transmissible [5], and that an additional helper plasmid is required for successful transfer. Similar results were found by Subbiah *et al*. for *E. coli* strain H4H [29]. This strain conjugated the pA/C (peH4H) at low frequency (10^-7^), yet when a DH10B strain harboring peH4H was used as donor no transconjugants were detected. When peH4H was combined with the H4H co-resident plasmid pTmpR in DH10B, however, transconjugants were obtained in the order of 10^-8^, suggesting that peH4H was mobilized by pTmpR in the wild-type strain. These investigators also found that 2/3 of the transconjugant population harbored either both plasmids or a large plasmid that presumably represented a chimera of these two plasmids [29]. We found that chimeric pA/C + pX1 were formed during *cis*-mobilization of YU39 pA/C by pX1. It seems that the pA/C lacks an *oriT* compatible with the conjugative type IV secretion systems of pX1, and when co-integrated with pX1 a successful transfer was achieved. Nevertheless, regardless of the recipient strain, in no case were the two plasmids observed in the transconjugant populations. These results suggest that the co-integrates were stable and not resolved; furthermore, these co-integrates maintained their architecture after a second round of conjugation.

### Acquisition of the CMY region by pX1

IncX plasmids have been less studied that IncA/C plasmids, but their record extends through the pre-antibiotic era [30]. Recent studies have focused on IncX because of their implication in biofilm formation and drug-resistance in *Enterobacteriaceae*[13,15,31]. In *Salmonella,* IncX plasmids have been related to co-integrates with serotype-specific virulence plasmids. pOG669 is a Typhimurium virulence plasmid co-integrated with an IncX conjugative plasmid carrying ampicillin and kanamycin resistance, which has been used in compatibility experiments among Typhimurium strains [32,33]. pOU1115 is a Dublin virulence plasmid that co-integrated with an IncX plasmid similar to pOU1114 [34]. In serovar Enteritidis, phage-type conversion has been demonstrated by the acquisition of IncX plasmids, such as pOG670 and pSE34 [35,36]. All IncX plasmids studied so far exhibit a type IV secretion system as part of their plasmid backbone [35,36]; this feature enables horizontal transfer of these plasmids between host cells. The ability to induce biofilm formation and the expression of conjugative type IV secretion systems could have a synergistic effect that ultimately could be related to the pathogenic potential of a bacterium [37]. YU39 pX1 was negative for the amplification of the biofilm-formation operon *mrk* (data not shown) characteristic of pX1 plasmids pMAS2027, pOLA52 and pLN126_33 [19]. However, the laboratory cultures of YU39 and its pX1 transformants and transconjugant exhibited a biofilm-formation-like halo, which could be the result of other fimbrial or outer membrane proteins carried by this plasmid. YU39 was originally isolated from an eight year old boy in Yucatán with a systemic infection that presented severe thrombocytopenia and active bleeding [4]. The contribution of pX1 to the pathogenic potential of YU39 will be addressed in further experimental research.

This is the first study to report the acquisition of an ESC-resistance gene by an IncX1 plasmid. The genetic contexts of *bla*_CMY-2_ genes have been addressed over the last decade [20,38-42]. The core CMY region is composed of a transposon-like element consisting of a specific IS*Ecp1*-*bla*_CMY-2_-*blc*-*sugE* structure. The genetic context of this structure varies in different plasmids, particularly for those genes downstream of *sugE*[20,39,41]. IS*Ecp1* codes for the transposase (*tnpA*) that mobilizes the CMY region by the one-end transposition mechanism, which only requires the action of one IS [43]. It is widely accepted that IS*Ecp1* may mobilize many antibiotic resistance genes by transposing adjacent to them, and then misreading its cognate ends during a second transposition event, with the effect of moving the adjacent genes [43]. The reports of variable surrounding regions of *bla*_CMY-2_ gene could be explained by the misreading end-effects of IS*Ecp1* and their movement among different genetic backgrounds. This is consistent with our results in which we found different versions of the original YU39 CMY region in the pX1*::*CMY transconjugant plasmids (short and large; Table 5). This variability may reflect the outcome of different one-end transposition events. Lartigue *et al*. reconstructed the process of mobilization of *bla*_CTX-M_ genes by IS*Ecp1B* in *Kluyvera ascorbata*[44]. They reported that IS*Ecp1B*-*bla*_CTX-M_ transposed at various insertion sites at frequencies of (6.4 ± 0.5) × 10^-7^. In all cases, genetic analysis of several transposition events revealed a 5-bp duplication that confirmed their acquisition by transposition [44]. No consensus sequence was identified among the 5 bp duplicated sites, whereas an AT-rich content that may target IS*Ecp1B*-mediated transposition was identified [44]. These results were highly similar to our own in which the calculated transposition frequency was in the range of 10^-6^ to 10^-9^ and the analysis of two pX1*::*CMY displayed different duplications at AT-rich regions, one of 5 bp and the other of 6 bp (Figure 2). Our results provide evidence of *in vivo* mobilization of a clinically important antibiotic resistance gene (*bla*_CMY-2_) from a non-conjugative pA/C to a highly conjugative pX1.

The insertion site for three pX1*::*CMY, carrying the “large” version of the CMY region, was the intergenic region between two ORFs with unknown function, here referred to as 046 and 047, based on the annotation of the reference plasmid pOU1114. This intergenic region is conserved in most of the sequenced IncX plasmids and is located in the region where the “genetic load” operons are frequently inserted (i. e. fimbrial or resistance genes) [19]. The insertion site for three pX1*::*CMY carrying the “short” version of the CMY region was *stbE*, which is the second gene of the *stbDE* operon involved in the plasmid addiction system. In toxin-antitoxin stability systems, the toxic activity of one protein is normally repressed by the partner antitoxin, when a plasmid-free variant arises, the antitoxin decays more rapidly than the toxin, and this releases the latter to act on its intracellular target, which results in cell death or stasis [45]. Therefore, inactivation of *stbE* toxin by the CMY region insertion was not lethal to the bacterial host. The fact that two pX1*::*CMY transconjugants for which the CMY insertion site could not be determined, evidence that other pX1 regions might be targets for IS*Ecp1* transposition. Our results suggest that transposition occurs more or less randomly in AT-rich regions of pX1, but only those not affecting replication and conjugation could be recovered in our conjugation experiments.

### Increased conjugation frequency of pA/C + pX1 and pX1::CMY

Our experiments demonstrate that YU39 pX1 conjugates at a very high frequency (10^-1^; Table 5). The pA/C + pX1 and pX1*::*CMY transconjugants also exhibited high frequencies when challenged for a second round of conjugation (Table 3 and Table 4). The differences in conjugation frequencies among pA/C + pX1 and pX1*::*CMY transconjugants with those of pX1, led us to determine that the transposition and co-integration events occurred within YU39 at frequencies ranging between 10^-6^ and 10^-9^, which were in the range of those reported for other transposition or co-integration events [18,43,44]. These results indicated that the first round conjugation frequencies combined the low frequency of co-integration or transposition with the high frequency of conjugation of pX1 (Table 5); while the second round conjugations directly measured the conjugation frequencies of pA/C + pX1 or pX1*::*CMY, which were high in most of the cases due to the use of the pX1 conjugative machinery (Table 3 and Table 4).

### *trans*-mobilization of pColE1-like

The mobilization capacities of ColE1 related plasmids have been recognized for decades, and plasmids from several incompatibility groups have been shown to mobilize them [46]. ColE1-like plasmids are prevalent in *Salmonella* serovars [11], and most of them carry the Km resistance gene *aph*[47,48].

The YU39 pColE1-like did not confer Km resistance nor to any other of the YU39 antibiotic resistances tested (data not shown). Despite the high frequency of transfer of the pColE1-like plasmids, our hybridization assays demonstrated that this plasmid was not involved in the genetic re-arrangements displayed by pA/C and pX1, or the acquisition of the *bla*_CMY-2_ gene. Taken together, these results suggest that pColE1-like is a very efficient molecular parasite. However, only the determination of its complete nucleotide sequence could provide information regarding the presence of a gene increasing the fitness of its host bacteria.

### Epidemiological implications

Our study demonstrated that pSTV and pA/C can indeed co-exist within *E. coli* and Typhimurium strains. Therefore, our original epidemiological observations that each of these plasmids was restricted to distinct genotypes [4] cannot be explained by negative interactions between them. In our previous studies we showed that the only strain capable of conjugative transfer of *bla*_CMY-2_ was YU39 [5]. We screened the Mexican population for the presence of pX1, but YU39 was the only positive strain (data not shown), explaining why the other ST213 pA/C lacked the capacity to be transferred. We hypothesize that pA/C emerged in ST213, which is a genotype lacking pSTV, and that the non-conjugative pA/C failed to colonize ST19 strains. The widespread dissemination of pA/C and *bla*_CMY-2_ in the ST213 population by the action of YU39 pX1 is a rare, but not negligible, event. Future epidemiological studies designed to track the prevalence of pX1 in the Mexican populations will shed light on these interactions.

## Conclusions

Our results provide evidence of *in vivo* mobilization of a clinically important antibiotic resistance gene (*bla*_CMY-2_) from a non-conjugative pA/C to a highly conjugative pX1. This work highlights the diverse possibilities that a single strain is capable to exploit, in order to contend with the challenge of horizontal gene transfer and antibiotic selective pressure.

## Competing interests

The authors declare that no competing interests exist.

## Authors’ contributions

MW conceived the study, performed most of the laboratory work, interpreted the data and drafted the manuscript. MFM designed the cloning and sequencing strategy for the IncX1 and ColE1-like plasmids and helped in the laboratory work. MAC participated in the design of the study, interpretation of data and helped to draft the manuscript. CZA performed the PCR screenings and helped in the laboratory work. MBZ provided the strains and drafted the manuscript. EC participated in the conception of the study, the interpretation of the data and helped to draft the manuscript. CS participated in the design of the study, performed part of the laboratory work, interpreted the data and drafted the manuscript. All authors read and approved the final manuscript.

## Supplementary Material

Additional file 1 **A) Plasmid profiles of the Typhimurium YU39 pA/C (*bla***_**CMY-2**_**) and SO1 pSTV*::Km *donors, and of the *E. coli *DH5α transformant strain carrying both plasmids.** B) The graphic depicts the stability of both plasmids in DH5α grown without antibiotic selection for up to 80 generations. The experiments were performed in triplicate. After incubation overnight at 37°C with shaking at 200 rpm, these cultures were washed twice to remove the antibiotics and re-suspended in 1 ml of 1 x PBS. From these cell suspensions, 100 μl were transferred to 100 ml LB without antibiotic and incubated with shaking for 24 hours at 37°C. The freshly inoculated cultures constituted time-point zero and the culture was estimated to have a cell density of about 3 × 10^6^ bacteria/ml by colony-count plating onto LB plates without antibiotics. Every 24 hours 100 μl of the full-grown cultures were transferred to fresh 100 ml LB without antibiotic and incubated with shaking at 37°C. Simultaneously, 100 μl of the full-grown cultures were diluted and plated onto LB plates without antibiotic. To determine the fraction of cells in the population harboring pA/C and pSTV*::Km* plasmids, 100 colonies from the LB plates were picked onto LB plates containing either CRO or Km. Two randomly chosen colonies were selected in all time points for pA/C and pSTV*::Km* PCR screening, with *repA/C*, R-7, *spvC* and *traT*. The number of generations was estimated by triplicate growth curves in 100 ml LB at 37°C with shaking at 200 rpm. Absorbance at 600 nm was recorded each hour. The growth rate was estimated from the equation: μ = log (t2/t1)/Ab2-Ab1; g = log2/μ: where t is the sample time in minutes, Ab is absorbance of the sample in t1 or t2, μ is duplication time and g is the growth rate constant. Click here for file

Additional file 2: Figure S2 Conjugation scheme. Typhimurium ST213 strain YU39 was used as donor of the *bla*_CMY-2_, gene (conferring resistance to ceftriaxone; CRO) carried by the pA/C plasmid. Five recipient strains were tested: two Typhimurium ST19 strains (SO1 pSTV*::Km* and LT2 pSTV*::Km*), and three *E. coli* strains (DH5α, HB101 and HB101pSTV*::Km*). The relevant plasmids are depicted by dotted circles (see text for details). Click here for file

Additional file 3: Table S1 Primers used in this study. Click here for file

Additional file 4: Figure S3 PCR typing scheme for pX1. The six regions used in the pX1 typing scheme are show on the sequence of the plasmid (unpublished data). The regions involved in plasmid replication *oriX* and *ydgA* are in blue; the regions involved in conjugation *taxB*, *taxC* and *ddp3* are in red; the intergenic region between 046-047 hypothetical protein genes and the *stbDE* operon were the CMY island was inserted (Figure 1) are in green. Click here for file
