# Supplementary figures and images for: Conjugative transfer of an IncA/C plasmid-borne blaCMY-2 gene through genetic re-arrangements with an IncX1 plasmid
Source: BMC Microbiol. 2013 Nov 21;13:264. doi: 10.1186/1471-2180-13-264 (PMC4222815; doi:10.1186/1471-2180-13-264)

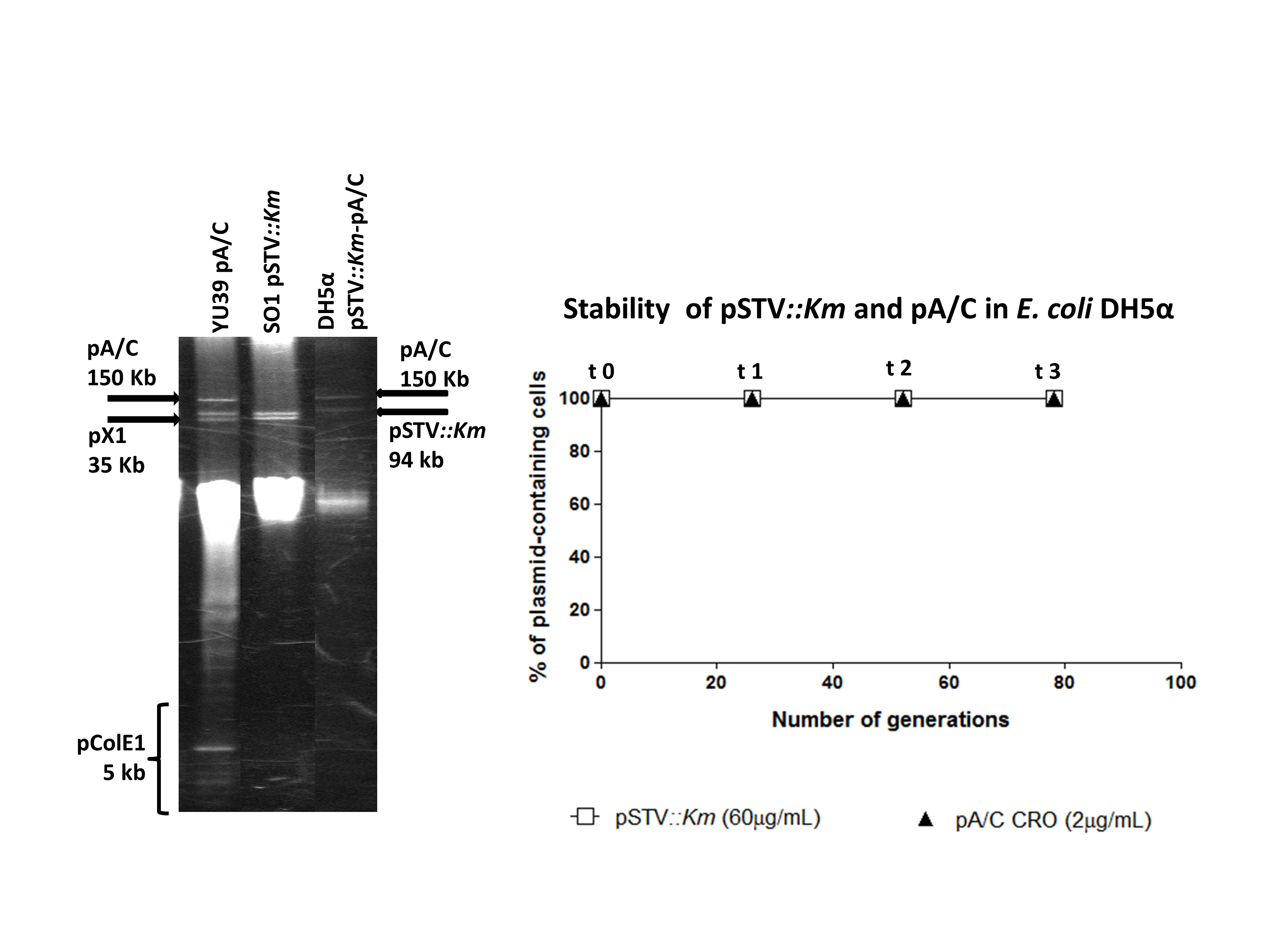

Supplement: Additional file 1 — A) Plasmid profiles of the Typhimurium YU39 pA/C (blaCMY-2) and SO1 pSTV::Km donors, and of the E. coli DH5α transformant strain carrying both plasmids. B) The graphic depicts the stability of both plasmids in DH5α grown without antibiotic selection for up to 80 generations. The experiments were performed in triplicate. After incubation overnight at 37°C with shaking at 200 rpm, these cultures were washed twice to remove the antibiotics and re-suspended in 1 ml of 1 x PBS. From these cell suspensions, 100 μl were transferred to 100 ml LB without antibiotic and incubated with shaking for 24 hours at 37°C. The freshly inoculated cultures constituted time-point zero and the culture was estimated to have a cell density of about 3 × 10^6^ bacteria/ml by colony-count plating onto LB plates without antibiotics. Every 24 hours 100 μl of the full-grown cultures were transferred to fresh 100 ml LB without antibiotic and incubated with shaking at 37°C. Simultaneously, 100 μl of the full-grown cultures were diluted and plated onto LB plates without antibiotic. To determine the fraction of cells in the population harboring pA/C and pSTV::Km plasmids, 100 colonies from the LB plates were picked onto LB plates containing either CRO or Km. Two randomly chosen colonies were selected in all time points for pA/C and pSTV::Km PCR screening, with repA/C, R-7, spvC and traT. The number of generations was estimated by triplicate growth curves in 100 ml LB at 37°C with shaking at 200 rpm. Absorbance at 600 nm was recorded each hour. The growth rate was estimated from the equation: μ = log (t2/t1)/Ab2-Ab1; g = log2/μ: where t is the sample time in minutes, Ab is absorbance of the sample in t1 or t2, μ is duplication time and g is the growth rate constant. [file 1471-2180-13-264-S1.tiff]

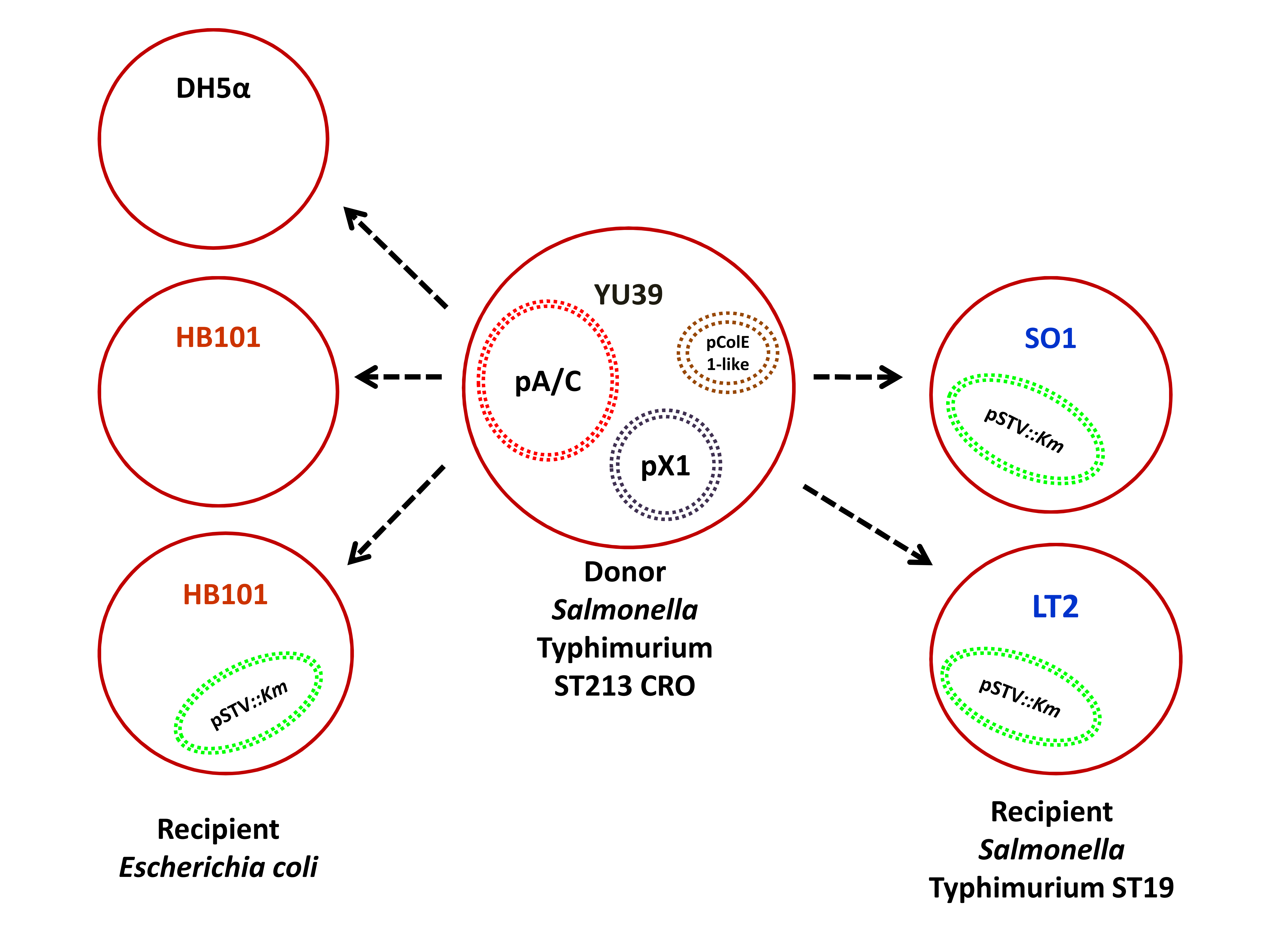

Supplement: Additional file 2: Figure S2 — Conjugation scheme. Typhimurium ST213 strain YU39 was used as donor of the blaCMY-2, gene (conferring resistance to ceftriaxone; CRO) carried by the pA/C plasmid. Five recipient strains were tested: two Typhimurium ST19 strains (SO1 pSTV::Km and LT2 pSTV::Km), and three E. coli strains (DH5α, HB101 and HB101pSTV::Km). The relevant plasmids are depicted by dotted circles (see text for details). [file 1471-2180-13-264-S2.tiff]

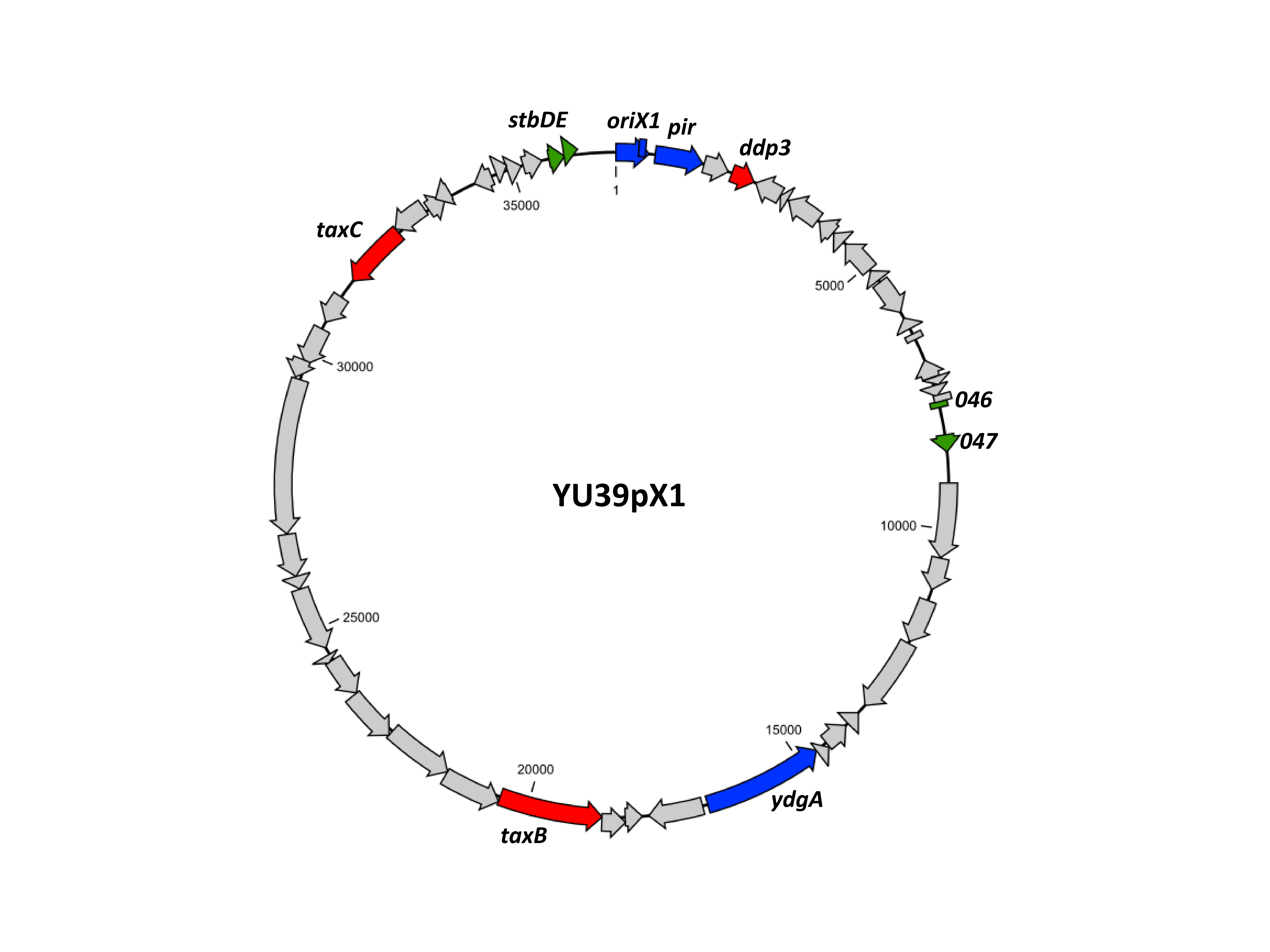

Supplement: Additional file 4: Figure S3 — PCR typing scheme for pX1. The six regions used in the pX1 typing scheme are show on the sequence of the plasmid (unpublished data). The regions involved in plasmid replication oriX and ydgA are in blue; the regions involved in conjugation taxB, taxC and ddp3 are in red; the intergenic region between 046-047 hypothetical protein genes and the stbDE operon were the CMY island was inserted (Figure 1) are in green. [file 1471-2180-13-264-S4.tiff]
