# Supplementary material for: Conjugative transfer of an IncA/C plasmid-borne blaCMY-2 gene through genetic re-arrangements with an IncX1 plasmid
Source: BMC Microbiol. 2013 Nov 21;13:264. doi: 10.1186/1471-2180-13-264 (PMC4222815; doi:10.1186/1471-2180-13-264)
Supplement: Additional file 3: Table S1 — Primers used in this study. [file 1471-2180-13-264-S3.doc]

Table S1. Primers used in this study

| **Assay** | **Primer** | **Gene** | **Sequence 5´- 3´** | **Size bp** | **Reference** |
| --- | --- | --- | --- | --- | --- |
| *spvC* mutagenesis | Spvh1p15´ | *spvC::Km* | AATTTCAACTCCTTGCACAACCAAATGCGGAAGATGCCGGTGTGTAGGCTGGAGCTGCTT | 1,500 | This study |
|  | Spvh2p23´ |  | TAAAGCCTGTCTCTGCATTTCGCCACCATCACGCCCACTTCATATGAATATCCTCCTTAGTTC |  |  |
| pSTV screening | SpvC-1 | *spvC* | ACTCCTTGCACAACCAAATGCGGA | 550 | Wiesner et al., 2009 |
|  | SpvC-2 |  | TGTCTCTGCATTTCGCCATCA |  |  |
|  | traT-F | *traT* | GATGGTTACACTGGTCAG | 500 | Wiesner et al., 2009 |
|  | traT-R |  | TCTGAGATCTGTACGTCG |  |  |
| pA/C screening | repA/C-F | *repA/C* | GAGAACCAAAGACAAAGACCTGGA | 300 | Welch et al., 2007 |
|  | repA/C-R |  | TTCTGGAGTTCGTACAGAGTGAAC |  |  |
|  | CMY-F | *cmy-2* | ATAACCACCCAGTCACGC | 600 | Wiesner et al., 2009 |
|  | CMY-R |  | CAGTAGCGAGACTGCGCA |  |  |
|  | R7-F | R-7 | CAGCACAAACATCTTCCCAGAC | 1,500 | Welch et al., 2007 |
|  | R7-R |  | GGGTAACACCGCCAACTCTTAC |  |  |
| pX1 screening | stbD5-F | *oriX1* | CTCATAAGCCCTCCGCTTGTCT | 2,000 | This study |
|  | pir3-R |  | TTTCACTGCAAAACATTTCTTACGC |  |  |
|  | DTOPOIII5-F | *ydgA* | AAAGACAAAAAAGATGAACATGGC | 800 | This study |
|  | HH3 II-R |  | TGATAAAGGCAGCTTGTAACGGT |  |  |
|  | taxC-F | *taxC* | CTGCGTAAACTGCGCCATTCGC | 1,000 | This study |
|  | taxC-R |  | AGTCAGCTTTCGCTCACAAAGTCA |  |  |
|  | taxB-F | *taxB* | TGTTGAGCGTCCCCGGCTCT | 1,500 | This study |
|  | taxB-R |  | CGATACTGATATTCTCGTCGGGGCA |  |  |
|  | ddp3-F | *ddp3* | CGTCTCTGACGCCATTCCGCA | 500 | This study |
|  | ddp3-R |  | TTTCCAGCTTATCGCTGGTAAGCAA |  |  |
| CMY insertion sites | pOU_46 | Intergenic 046-047 | TACGCTGCCTGTCACCACGG | 600 | This study |
|  | pOU_47 |  | TCTCAGGGCTGTGCTCGCCA |  |  |
|  | stbE-F | *stbDE* | ATTCTGACAACCACAGCGGCCAG | 500 | This study |
|  | stbD-R |  | CTGCGATCTGATAGACTTCGCTGCT |  |  |
| pX1-CMY junction | stbE-F | CMY-*stbED* junction | ATTCTGACAACCACAGCGGCCAG | 1,000 | This study |
|  | tnpA2-L |  | TAGCCACTATCCATTCGAAATAAAA |  | Wiesner et al., 2009 |
|  | sugE-F |  | GGGTGAAACAGCCTGGCGCT | 800 | Wiesner et al., 2009 |
|  | stbD-R |  | CTGCGATCTGATAGACTTCGCTGCT |  | This study |
|  | pOU_47 | CMY-046-047 junction | TCTCAGGGCTGTGCTCGCCA | 1,600 | This study |
|  | CMY-R |  | CAGTAGCGAGACTGCGCA |  | Wiesner et al., 2009 |
|  | pOU_46 |  | TACGCTGCCTGTCACCACGG | 1,000 | This study |
|  | Hyp-F2 |  | TTCCAAGGTAAAAGTTGGGTCTG |  | Wiesner et al., 2009 |
| pX1 *taxB* mutant | TaxB_p1 | *taxB* | tgactatttcagaaaactgaaggagtgattatgtctttaaaactcccaTGTAGGCTGGAGCTGCTTCG | 1,500 | This study |
|  | TaxB_p2 |  | ggaaaagaatactgatcataagtatttttttcataataaaacctcagaCATATGAATATCTCTTTAG |  |  |
| pColE1-like screening | mobA-F | *mobA* | GATCAGTTTCG CACGTTCCAG | 1,570 | This study |
|  | mobA-R |  | GGGAACTCATTGATGCCACG |  |  |
| pUC18 clones | lacZ-F | *lacZ* | GGTTTTCCCAGTCACGACGTT | Variable |  |
|  | lacZ-R |  | GAATTGTGAGCGGATAACAATTTC |  |  |

1. Wiesner M, Zaidi MB, Calva E, Fernandez-Mora M, Calva JJ, Silva C: **Association of virulence plasmid and antibiotic resistance determinants with chromosomal multilocus genotypes in Mexican *Salmonella enterica* serovar Typhimurium strains.** *BMC Microbiol* 2009, **9:**131.

2. Welch TJ, Fricke WF, McDermott PF, White DG, Rosso ML, Rasko DA, Mammel MK, Eppinger M, Rosovitz MJ, Wagner D, et al: **Multiple antimicrobial resistance in plague: an emerging public health risk**. *PLoS ONE* 2007, 2:e309.
